# In Vitro Effects of Cypermethrin and Glyphosate on LPS-Induced Immune Cell Activation

**DOI:** 10.3390/life14010062

**Published:** 2023-12-29

**Authors:** Narjesse El Mabrouk, Martina Iulini, Ambra Maddalon, Valentina Galbiati, Hedi Harizi, Maha Mastouri, Emanuela Corsini

**Affiliations:** 1 Laboratory of Transmissible Diseases and Biologically Active Substances, Faculty of Pharmacy, Monastir University, Avenue Avicienne, Monastir 5019, Tunisia; narjesse.elmabrouk@fst.utm.tn (N.E.M.); hedi.harizi@fmdm.u-monastir.tn (H.H.); maha.mastouri@rns.tn (M.M.); 2 Laboratory of Toxicology, Department of Pharmacological and Biomolecular Sciences ‘Rodolfo Paoletti’, University of Milan, Via Balzaretti 9, 20133 Milan, Italy; martina.iulini@unimi.it (M.I.); ambra.maddalon@unimi.it (A.M.); emanuela.corsini@unimi.it (E.C.)

**Keywords:** pesticides, immunotoxicity, in vitro method, cytokines, surface markers, new approach methodologies

## Abstract

(1) Background: The insecticide cypermethrin (Cypm) and the herbicide glyphosate (Glyp) are among the most widely used pesticides. While the two pesticides have been considered to have low toxicity in mammals, some indication of potential immunotoxicity has emerged. The aim of this work was to investigate in vitro the effects of Cypm and Glyp on bacteria lipopolysaccharide (LPS)-induced immune cell activation and of Cypm on 2-mercaptobenzothiazole (MBT)-induced maturation of dendritic cells (DCs). (2) Methods: The release of the inflammatory cytokines TNF-α and IL-8, the expression of the surface markers CD54 and CD86 in human primary peripheral blood mononuclear cells (PBMC), and THP-1 cells were investigated together with CD83, HLA-DR, IL-6, and IL-18 in DCs. (3) Results: While no significant modulation on LPS-induced immune cell activation was observed following Glyp exposure, with only a trend toward an increase at the highest concentration tested, Cypm reduced the responses to LPS and to MBT, supporting a direct immunosuppressive effect. Overall, the present study contributes to our understanding of pesticide-induced immunotoxicity, and the results obtained support evidence showing the immunosuppressive effects of Cypm.

## 1. Introduction

Despite an undisputed utility in the use of pesticides in agriculture and in the protection of public health, their indiscriminate and excessive use are considered worldwide a major public health problem because of their potential harmful effects on humans and environment [1,2,3,4]. The immune system is one potential sensitive target of toxicity following exposure to variety of xenobiotics, including pesticides [5,6]. In the last decades, industrialized countries faced an increase in diseases, like cancer, hypersensitivity, and autoimmunity, attributable to an alteration of the immune system function, and concern is growing that this trend could be at least partially attributable to new and modified patterns of exposure to chemicals, including pesticides [7,8,9,10]. Pesticides are chemical compounds commonly and heavily employed in modern agriculture, households, forestry, horticulture, industry, medicine, and veterinary medicine [2]. Based on their specific chemical structure and target, pesticides can be classified into various classes, including insecticides and herbicides. Their pervasive worldwide use has resulted in environmental contaminations [11], as well as the constant exposure to human and animals, thus becoming a focus of global concern [1]. 

Pyrethroids are one of the most widely used pesticides because they are considered of low toxicity, of low tendency to accumulate in organisms, and with a short biodegradation period compared to other kinds of insecticides [12,13,14]. Cypermethrin (Cypm) is a class II pyrethroid pesticide used to control insects in household and agricultural fields. As a mechanism of action, Cypm prolongs the opening of sodium channels; modulates chloride, voltage-gated calcium, and potassium channels; and alters the activity of glutamate and acetylcholine receptors and the level of neurotransmitters, including gamma-aminobutyric acid and dopamine [4]. Recent data showed that pyrethroids can be detected in liquid human secretions as well as breast milk and urine [15,16]. Humans can be exposed to pyrethroids by their use in personal protection (mosquito nets), disinfection in aircrafts, agriculture, etc. Emerging data indicate that they are not completely harmless to human health, potentially affecting fertility, the immune system, and cardiovascular and hepatic metabolism [17].

Even if still controversial, several studies indicated that pyrethroids have the potential to cause immunotoxicity. The immunomodulatory effects of Cypm have been demonstrated in mammals, in which exposure at doses higher than 10 mg/kg × body weight resulted in immunosuppression. Immunosuppression was found at doses not showing toxic clinical signs or behavioral changes [18,19,20,21]. Dési et al. (1986) [18] reported in rats and rabbits a dose-dependent suppression of humoral and cell mediate immune responses. Varshneya et al. (1992) [19] reported leucopenia, a decrease in spleen weight, and a dose-dependent decrease in delayed-type hypersensitivity reaction following 90 day exposure to Cypm. Tamang et al. (1988) [20] investigated in mice and goats the effects of Cypm following intraperitoneal injection, wherein a significant reduction in 4-dinitrofluorobenzene skin sensitivity test in mice and a decrease in plaque-forming B-lymphocytes in goats were found. Institóris et al. (2002) [21] investigated the effect of Cypm in Wistar rats following oral exposure, wherein 4 week oral treatment with 55.4 mg/kg suppressed both humoral (IgM-PFC following sheep red blood cells immunization) and cellular (delayed type hypersensitivity reaction to keyhole limpet hemocyanin) immune responses. Alterations in immunological parameters were also observed in humans [22]. Exposed greenhouse workers showed a significant decrease in plasma IL-12p70, IFN-γ, IL-2, and IL-8 [22]. In vitro studies also support Cypm immunosuppression, indicating a direct effect of Cypm on immune cells. Stelzer and Gordon (1984) [23] showed that Cypm inhibited the proliferation of mouse T and B cells to concanavalin A and lipopolysaccharide (LPS) at the 10–50 mM concentration range. Wang et al. (2017) [24] reported in the mouse macrophage cell line RAW 264.7 the ability of Cypm to inhibit mRNA levels of pro-inflammatory cytokines (IL-1β, IL-6, CXCL-1, TNF-α, IFN-γ) with or without LPS stimulation, as well as phagocytosis. More recently, we also observed in THP-1 cells a decrease in LPS-induced IL-8, TNF-α, CD86, and CD54 after Cypm exposure [25]. 

Similarly, the herbicide glyphosate (Glyp) (N-phosphomethyl [glycine]) is a widely used pesticide, considered the most used broad-spectrum herbicide worldwide [26]. Glyp has longtime been regarded as harmless because it targets the enzyme 5-enolpyruvylshikimate-3-phosphate synthase and blocks the production of chorismite, an essential intermediate in the shikimate pathway responsible for the biosynthesis of aromatic amino acids in plants and microorganisms, which is absent in mammals. Currently, the use of Glyp is very debated, as several studies indicating its hazard were emerging, and Glyp is classified as “probably carcinogenic” in humans by the International Agency for Research on Cancer (IARC) [27]. In the EU, the renewal of its authorization was approved and renewed in November 2023 (EU 2023/2660).

Concerning immunotoxicity of Glyp, there was a limited number of original in vitro and in vivo papers published and, even if limited in number, they pointed out possible adverse effects of glyphosate on the immune system, with both immunosuppression and inappropriate immunostimulant observed, which is not contradictory considering the nature of the immune system and the underlying mechanisms of immune activation [27,28]. We recently investigated the effect of Glyp on T helper (Th) cell differentiation and cytokine production. A reduction of Th1/Th2 ratio, mainly due to a decrease in Th1 cells, was observed following Glyp exposure, together with an enhancement of IL-4 and IL-17A production, as well as a reduction of IFN-γ. Based on literature evidence that suggested Glyp being an endocrine disruptor, we also demonstrated a role of ER in the observed effects [29].

Based on our previous data showing immunomodulatory effects of both Cypm and Glyp, the purpose of this study was to further address their immunotoxic potential. The effects of Cypm and Glyp on LPS-induced immune activation in human primary peripheral blood mononuclear cells (PBMC) and in the human leukemia monocytic cell line THP-1, as well as the effect of Cypm on 2-mercaptobenzothiazole (MBT)-induced dendritic cell (DC) maturation, were investigated. As immune activators, LPS (endotoxin), the principal structure of Gram-negative bacteria, and MBT were used. LPS is a prototypical immune system activator, mainly of the cells of the myeloid lineage, triggering numerous immunostimulatory effects, and at high does leading to septic shock [30]. The rubber accelerator MBT, known to cause allergic contact dermatitis, was used as a reference compound to activate DC maturation [31]. As markers of activation, both surface markers (e.g., CD54, CD86) and cytokine release (e.g., IL-8, TNF-α) were measured.

## 2. Materials and Methods

### 2.1. Chemicals and Reagents

Cypm (CAS #52315-07-8, purity ≥ 99%) was purchased from Sigma-Aldrich (St. Louis, MO, USA), and it was dissolved in dimethyl sulfoxide (DMSO, CAS #67-68-5, purity ≥ 99.5%, Sigma-Aldrich). Glyp (CAS #1071-83-6, purity ≥ 99%) was purchased from Sigma-Aldrich, and it was dissolved in Dulbecco’s phosphate-buffered saline (PBS, Sigma-Aldrich). The allergen MBT (CAS #149-30-4, purity 97%) and the lipopolysaccharide from *Escherichia coli* serotype O27:B8 (LPS) were purchased from Sigma-Aldrich and dissolved in PBS. Cell culture media and all supplements were from Sigma-Aldrich. All reagents were purchased at the highest purity available.

### 2.2. Cells

PBMC were obtained by Ficoll gradient centrifugation from anonymous buffy coats of 5 male healthy donors, purchased from the Niguarda Hospital in Milan (Italy). The present study was conducted using only male donors, starting from the occupational notion that in Italy, the majority of pesticide applicators are males. After centrifugation, PBMC layers were removed and were washed 5 times with PBS. Isolated cells were diluted to 10^6^ cells/mL in RPMI 1640 containing 2 mM L-glutamine, 0.1 mg/mL streptomycin, 100 IU/mL penicillin, 10 μg/mL gentamycin, and 50 μM 2-mercaptoethanol, supplemented with 10% heated-inactivated fetal bovine serum (culture media) and cultured at 37 °C in a humidified atmosphere of a 5% CO_2_ incubator.

The THP-1 cell lines (Elabscience Biotechnology Inc.—Houston, TX, USA) were cultured at 10^6^ cells/mL in RPMI 1640 containing 2 mM L-glutamine, 0.1 mg/mL streptomycin, 100 IU/mL penicillin, 10 μg/mL gentamycin, and 50 μM 2-mercaptoethanol, supplemented with 10% heated-inactivated fetal bovine serum (culture media) and cultured at 37 °C in a humidified atmosphere of a 5% CO_2_ incubator.

To obtain immature DCs (iDCs), THP-1 cells were differentiated with 100 ng/mL of rhIL-4 and 100 ng/mL of rhGM-CSF (ImmunoTools, Friesoythe, Germany) for 5 days. In these 5 days, the medium was change twice, and the differentiating factors were added each time. At the end of the 5 days, the differentiation was checked by FACS analyses (see below), and cells were used for the experiment.

All experiments with Cypm and Glyp were performed using RPMI 1640 without phenol red containing 2 mM L-glutamine, 0.1 mg/mL streptomycin, 100 IU/mL penicillin, 10 μg/mL gentamycin, and 50 μM 2-mercaptoethanol, supplemented with 5% heat-inactivated dialyzed fetal bovine serum. Preliminary experiments were conducted to identify non-cytotoxic concentrations (cell viability > 90%). Cytotoxicity was assessed with propidium iodide (PI) staining (Sigma-Aldrich).

### 2.3. Cells Treatment

Three different cell treatments were performed. The first one involved the use of LPS as a stimulus, while the other two involved the use of the allergen MBT.

Concerning the first, PBMC (10^6^ cells/mL) were incubated overnight and then treated with Cypm and Glyp, while THP-1 cells (10^6^ cells/mL) once diluted were treated immediately in the presence or absence of pesticides. In both models, cells were treated with increasing concentrations of Cypm (0.04, 0.4, and 4 μg/mL) or of G (0.01, 0.1, and 1 μg/mL). After 24 h, PBMC cells were stimulated with LPS (100 ng/mL), whereas THP-1 cells were stimulated with 10 ng/mL (for CD86 analysis) or 1 ng/mL (for CD54 analysis) of LPS for an additional 24 h. LPS concentrations were selected based on previous experiments as suitable to highlight differences in the response to LPS. At the end of treatment, supernatants were used to measure the release of cytokines while cells were used for flow cytometer analyses.

The other two treatments involved the use MBT, naïve THP-1, and iDCs. Naïve THP-1 (10^6^ cells/ mL) was treated in the presence or absence of Cypm at the concentrations of 0.4 μg/mL. After 24 h of Cypm exposure, cells were stimulated with MBT (30 μg/mL) for an additional 24 h. At the end of treatment, supernatants were used to measure the release of cytokines while cells were used for flow cytometer analyses. iDCs (2 × 10^5^ cells/mL), obtained as described above, were treated with DMSO as a negative control, MBT alone (30 μg/mL), C (0.4 μg/mL) and MBT together, or with the maturation cocktail to obtain mature DCs (mDCs) (200 ng/mL of rhIL-4, 100 ng/mL of rhGM-CSF, 20 ng/mL of rhTNF-α (CAS# 94948-59-1, Sigma-Aldrich), and 200 ng/mL of ionomycin (CAS# 56092-81-0, Sigma-Aldrich)). After 72 h, the treatment was stopped, and supernatants were used to measure the release of cytokines while cells were used for flow cytometer analyses.

### 2.4. Flow Cytometric Analysis

After treatment, cells were centrifuged at 0.3 g for 5 min and stained with the different markers. Specific FITC-conjugated antibody against human CD40, PE-conjugated antibody against human CD54, specific FITC-conjugated antibody against human CD80, specific PE-conjugated antibody against human CD83, specific FITC-conjugated antibody against human CD86, and specific FITC-conjugated antibody against human HLA-DR or with isotype control antibody (BD Biosciences, Franklin Lakes, NJ, USA) were used. All of the antibodies selected have the mouse IgG1 κ isotype. Following the manufacturer’s instructions for each sample, 3 μL of CD40, CD80, CD83, CD86, and HLA-DR were added in 200 μL of PBS, while for CD54 and isotype control, 1.5 μL was added into 200 μL of PBS. To assess the proper differentiation from naïve THP-1 to iDCs, CD40, CD80, CD86, and related isotypes were used. To study the effect of Cymp and Glyp on LPS-induced maturation, CD54 and CD86 were used, while to study the effect of Cypm on MBT-induced cell activation, CD83, CD86, and HLA-DR were measured.

Cells were analyzed using a NovoCyte 3000 flow cytometer, and data were quantified using NovoExpress software Version 1.6.2 (Acea Biosciences, Inc., Santa Clara, CA, USA). A total of 10,000 viable cells were analyzed for mean fluorescence intensity (MFI). The MFI of isotype control was subtracted from the MFI of specific surface-marker-stained cells. Changes in surface marker expression are reported as stimulation index (SI) calculated by the following equation:

SI = MFIt/MFIc

MFIt stands for chemical-treated cells, whereas MFIc represents the untreated ones.

### 2.5. Cytokine Release

After treatment, the release of cytokine was assessed in cell-free supernatants following centrifugation at 0.2 g for 5 min. Cytokine production was assessed using specific sandwich enzyme-linked immunosorbent assays (ELISAs) that are commercially available. ELISAs for IL-6 and IL-8 were purchased from ImmunoTools GmbH (Friesoythe; Germany); IL-18 was purchased from Medical & Biological Laboratories (Terasawaoka Ina, Nagano, Japan); and human TNF-α was purchased from R&D Systems, Inc. (Minneapolis, MN, USA). Antibodies dilutions were performed according to the manufacturer’s instructions. Limits of detection were 2.6 pg/mL for IL-8, 6.1 pg/mL for IL-6, and 15.6 pg/mL for IL-18 and TNF-α. Results are expressed as fold-change (SI) of released cytokines of pesticide-treated versus control cells.

### 2.6. Data Analysis

All PBMC experiments were conducted using 5 donors. For THP-1 experiments, three independent experiments were conducted. Data are expressed as mean ± standard error of the mean (SEM). Statistical analysis was performed using GraphPad Prism version 9.1.1 (GraphPad Software, La Jolla, CA, USA). Data were analyzed using one-way analysis of variance (ANOVA), followed by Dunnett’s multiple comparison test or paired Student’s *t* test as indicated in the legends. Differences were considered significant at *p* ≤ 0.05.

## 3. Results

### 3.1. The Effects of Cypm on CD86, CD54 Expression, and IL-8 and TNF-α Release in PBMC

**Figure 1:**
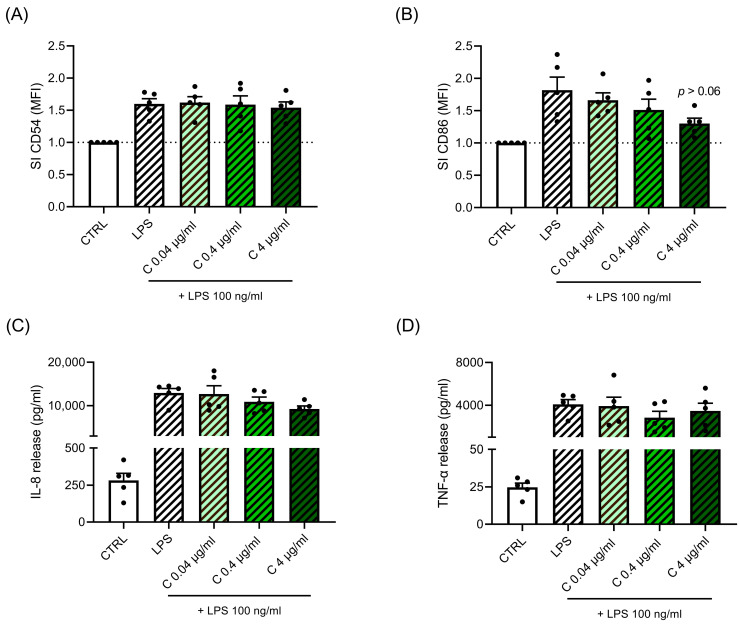
Effects of Cypm on LPS-induced CD54 and CD86 expression, and on TNF-α and IL-8 production. PBMC (10^6^ cells/mL) were treated for 24 h with increasing concentrations of Cypm (0.04, 0.4, 4 μg/mL) and then stimulated with 100 ng/mL of LPS for 24 h, as described in the Section 2. CD54 (**A**) and CD86 (**B**) expressions, as evaluated by FACS analysis, are expressed as fold-change (SI) of MFI of DMSO-treated cells. Cytokines were measured by ELISA in cell-free supernatants. The results of IL-8 (**C**) and TNF-α release (**D**) are expressed as fold change (SI) of the cytokine released in DMSO-treated cells The dotted line reported is set at 1.0 (DMSO control, CTRL). Each value represented the mean ± SEM, *n* = 5 donors. Data were analyzed by ANOVA followed by Dunnett’s multiple comparison test.

We previously demonstrated in THP-1 cells that Cypm inhibited LPS-induced cell activation as assessed by CD54, CD86 expression, and IL-8 and TNF-α release, reaching statistical significance at 4 μg/mL [25]. To confirm these results in primary human cells, PBMC obtained from healthy male donors (*n* = 5) were exposed for 24 h to increasing non-cytotoxic concentrations of Cypm (0.04–4 μg/mL) or DMSO as vehicle control, and then to LPS (100 ng/mL) for a further 24 h. Cell viability was higher than 90%, as assessed by PI staining. As shown in Figure 1, no changes in LPS-induced CD54 expression (Figure 1A) and TNF-α release (Figure 1D) were observed in PBMC, while Cypm induced a dose-related decrease in LPS-induced CD86 (Figure 1B) and IL-8 release (Figure 1C), which reached almost a statistical significance at 4 μg/mL (10 μM) (*p* < 0.06), consistent with the results obtained in THP-1 cells. This discrepancy may be explained by the lower LPS concentrations used in THP-1 cells (1 ng/mL for CD54 and 10 ng/mL for TNF-α, which being suboptimal, may allow for the highlighting of differences in the response masked using higher LPS concentrations). Further experiments in PBMC using lower LPS concentrations should be conducted to confirm these hypotheses.

### 3.2. Effect of Cypm on MBT-Induced DC Maturation

**Figure 2:**
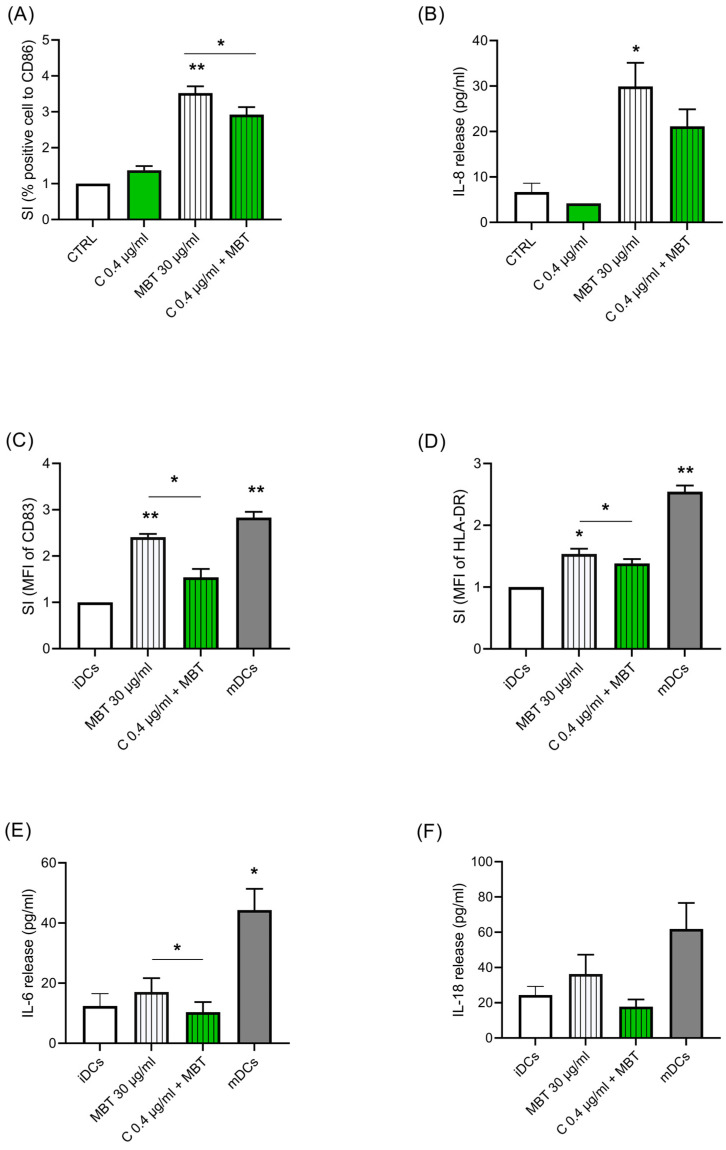
Effects of Cypm (reported as C in the graph) on MBT-induced activation in naïve THP-1 and iDCs. (**A**,**B**) Naïve THP-1 cells (10^6^ cells/mL) were treated for 24 h with Cypm (0.4 μg/mL) or with DMSO as vehicle, then stimulated with or without MBT (30 μg/mL) for 24 h. The expression of CD86 (**A**) and the release of IL-8 (**B**), as expressed as pg/mL, are reported. (**C**–**F**) iDCs (2 × 10^5^ cells/mL) were treated for 72 h with Cypm (0.4 μg/mL) plus MBT (30 μg/mL), MBT alone, or with maturation cocktail (rhIL-4, rhGM-CSF, rhTNF-α, and ionomycin) (mDCs), as described in Section 2. The expression of CD83^+^ cells (**C**) and HLA-DR^+^ cells (**D**) as expressed as fold-change (SI) were measured by FACS analysis, and the release of IL-6 (**E**) and IL-18 (**F**), expressed as pg/mL, was measured by ELISA. Each value represents the mean ± SEM, *n* = 3 independent experiments. Data were analyzed by paired *t*-test, with * *p* < 0.05 and ** *p* < 0.01.

As DCs are the bridging elements between innate and adaptative immune responses, we next investigated the biological alterations triggered by Cypm in naïve THP-1, and in the DC model, THP-1 following exposure to the contact allergen MBT. First, we investigated the modulation of Cypm on MBT (30 μg/mL)-induced naïve THP-1 activation, as assessed by CD86 expression and IL-8 release (Figure 2A,B). These two markers were chosen as they are selectively induced by chemical allergens [32]. As expected, MBT upregulated both markers. The treatment with Cypm (0.4 μg/mL) reduced both MBT-induced CD86 in a statistically significant way and sleekly also the release of IL-8, similarly to what was previously observed in response to LPS [25]. Then, to further substantiate this finding, the ability of Cypm to modulate DC maturation was investigated. The DC model THP-1 was used, as these cells represent an established model to study DCs, as they differentiate rapidly into mDCs when cultured in the presence of GM-CSF, TNF-α, and ionomycin [33]. iDCs were treated for 72 h with Cypm at the concentration of 0.4 μg/mL in the presence of MBT (30 μg/mL). The cytokine maturation cocktail (rhIL-4, rhGM-CSF, rhTNF-α, and ionomycin) (mDCs) was used as a positive control. The expressions of CD83 (Figure 2C) and HLA-DR (Figure 2D), as well as the release of IL-6 (Figure 2E) and IL-18 (Figure 2F), were measured as markers of DC activation. As expected, MBT alone and the maturation cocktail (mDCs) upregulated all markers, while the presence of Cypm significantly reduced all maturation markers (i.e., CD83, HLA-DR, IL-6, IL-18), indicating an immunosuppressive effect also at the level of DC activation. Cypm alone had no effect on any of the parameters measured.

### 3.3. Effect of Glyp on LPS-Induced Immune Cell Activation

**Figure 3:**
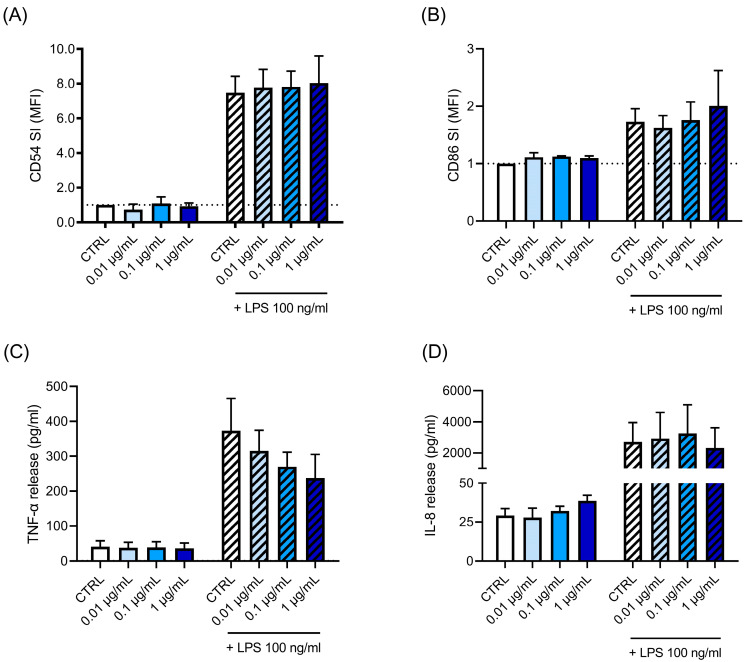
Effects of Glyp on LPS-induced CD54 and CD86 expression, as well as TNF-α and IL-8 release in THP-1 cells. THP-1 (10^6^ cells/mL) was treated for 24 h with increasing concentrations of Glyp (0.01, 0.1, and 1 μg/mL) and then stimulated for 24 h with LPS (100 ng/mL). (**A**) CD54; (**B**) CD86; (**C**) IL-8; (**D**) TNF-α. The expression of CD86 (**A**) and CD54 (**B**) was reported as fold change of the number of positive cells in Glyp-treated cells compared to control cells. Cytokine production was measured by ELISA in cell-free supernatants and expressed as pg/mL. The dotted line reported in (**A**,**B**) is set at 1.0 (DMSO control, CTRL). Each value represents the mean ± SEM, *n* = 3 independent experiments. Data were analyzed by ANOVA followed by Dunnett’s multiple comparison test.

**Figure 4:**
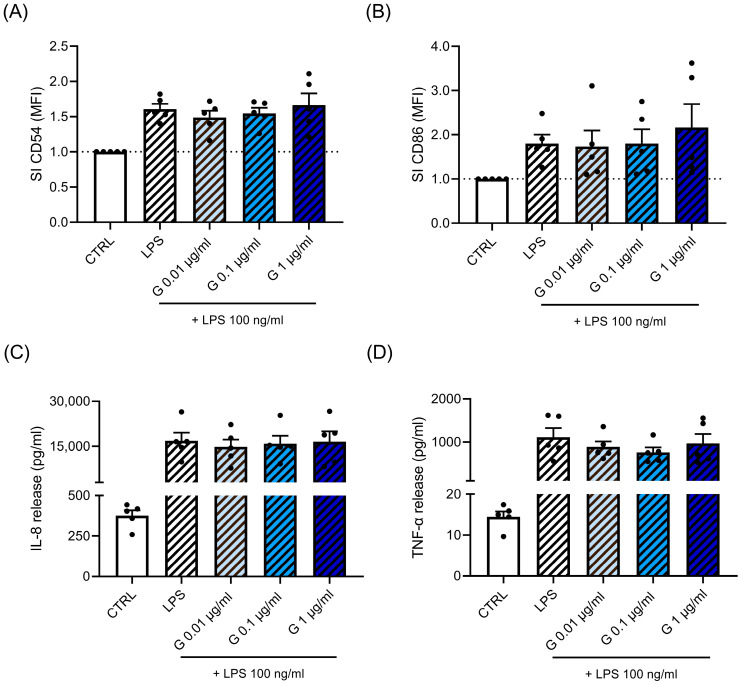
Effects of Glyp on LPS-induced CD54 and CD86 expression and TNF-α and IL-8 production. PBMC (10^6^ cells/mL) were treated for 24 h with increasing concentrations of Glyp (0.01, 0.1, and 1 μg/mL) and then stimulated with 100 ng/mL of LPS for 24 h, as described in the Section 2. CD54 (**A**) and CD86 (**B**) expressions, as evaluated using FACS analysis, are expressed as fold-change (SI) of MFI of DMSO-treated cells. Cytokines were measured by ELISA in cell-free supernatants. The results of IL-8 (**C**) and TNF-α release (**D**) are expressed as pg/mL of the cytokine released. For (**A**,**B**), the dotted line reported is set at 1.0 (DMSO control, CTRL). Each value represents the mean ± SEM, *n* = 5 donors. Data were analyzed by paired *t*-test.

In parallel to Cypm, we investigated the effect of Glyp on LPS-induced immune cell activation. The effect of Glyp was assessed both in THP-1 cells (Figure 3) and PBMC (Figure 4). As parameters of activation, CD54, CD86, IL-8, and TNF-α were measured. THP-1 cells were treated with increasing concentrations of Glyp (0.01–1 μg/mL) for 24 h and then stimulated with LPS (100 ng/mL) for a further 24 h. CD54- and CD86-positive cells were analyzed using flow cytometry (Figure 3A,B, respectively), while IL-8 and TNF-α were measured using ELISA (Figure 3C,D, respectively). No statistically significant effects were found in all the parameters investigated. Similar results were observed in PBMC (Figure 4). Only, a slight upward trend was observed at the higher concentration tested (1 μg/mL) for CD86, for which in two donors’ higher expression was observed. Overall, the data did not suggest immunomodulation following LPS activation, but rather, if at all, immunostimulation may be the consequence of concomitant Glyp exposure.

## 4. Discussion

Exposure to pesticides has been associated with negative effects on both innate and adaptive immunity, promoting deregulation in innate, humoral, and cellular processes, including cytokine expression, antibody production, and cell proliferation and differentiation, which are crucial mechanisms for host defense against pathogens and transformed cells [7,9,28,34,35,36]. Based on our previous data, showing immunomodulatory effects of both Cypm and Glyp, the purpose of this study was to further characterize their immunotoxic potential. In particular, the effects on LPS-induced immune activation in human primary PBMC and on MBT-induced DC maturation were investigated. While no significant modulation on LPS-induced immune cell activation was found following Glyp exposure under conditions in these in vitro studies, Cypm reduced the responses to LPS and MBT, supporting direct immunosuppressive effects.

In response to antigen or endotoxin stimulation, the innate immune system reacts by expressing surface markers and producing several mediators like chemokines, cytokines, and prostaglandins that lead to the activation of adaptive immune response and eventually to the eradication of the pathogen [37]. Cytokines have been considered to be important markers to assess the potential immunotoxicity of xenobiotics and the control of immune responses. They are involved in the regulation of many essential processes, including inflammation, apoptosis, and hematopoiesis [38]. Similarly, surface markers are central in the initiation of innate and adaptive immune responses. The adhesion marker CD54, known as ICAM-1, contributes to different processes such as inflammation, recruitment of immune cells, and antigen recognition. Likewise, CD86 expression modulates in a fine way and represents a critical checkpoint in the initiation of the adaptive immune response. In fact, CD86 is able to interact with CD28, leading to the activation and proliferation of T cells, or CTLA-4 resulting in an inhibition of the adaptive immune response. Only few studies have investigated the effects of Cypm and Glyp on inflammatory cytokine release and cell surface marker expression.

The concentrations used in the current study should be considered relevant for human exposure. For instance, the concentration of 0.1 μg/mL of Glyp is biologically relevant as serum levels ranging 0.2–189.1 ng/mL were detected in Thai women involved in agricultural activities [39]. For Cypm, human plasma levels are seldom available due to rapid metabolism and urinary excretion. Appenzeller et al. (2017) [40], comparing hair, urinary, and plasma levels of several pesticides, reported in rats exposed to Cypm 10 μg/kg an average plasma level of 0.25–43.4 ng/mL. In addition, considering the overlapping effects observed on plasma cytokine levels in greenhouse workers exposed to C and our in vitro results, this indicates the relevance of the concentration used as similar effects were observed [22].

Concerning immunotoxicity, as described in Section 1, several studies conducted in animal models indicated that exposure to Cypm, at doses > 10 mg/kg × body weight, was mainly associated with immunosuppression (e.g., decrease in delayed type hypersensitivity, leucopenia, decreased T-cell-dependent antibody response) [18,19,20,21]. It is important to note that these effects were observed in the absence of general toxicity or behavioral changes, indicating that the immune system may be a sensitive target of Cypm. The decrease in the response to LPS or MBT observed in our study is in agreement with the abovementioned in vivo immunosuppressive findings. The evidence of changes in immunological parameters have also been reported in humans [22]. Literature research highlighted the immunotoxicity of Cypm, assessed in occupationally exposed greenhouse workers [41]. Exposed workers showed neither clinical signs of immunosuppression nor alterations in total leukocytes or leukocyte subpopulations, whereas significant differences were found for IL-2, IL-8, IL-12p70, and IFN-γ. The decrease in IL-8, and the lack of effect on TNF-α, are consistent with our finding in PBMC. The decrease in type 1 cytokines (e.g., IL-2, IL-12p70, and IFN-γ) may lead to decreased cell-mediated immunity, which together with a decrease in pro-inflammatory cytokines (e.g., IL-8) may result in increased risk of infection and cancer [41,42].

DCs are responsible for initiating all antigen-specific immune responses. As such, they are the master regulators of the immune response and link the microbial sensing features of the innate immune system to the exquisite specificity of the adaptive response, both humoral and cell-mediated [43]. The ability of Cypm to modulate LPS-induced activation and MBT-induced DC activation should be considered detrimental to host resistance, reducing the ability to combat infections and diseases, including cancer [44]. While evidence of an increased risk of infection following exposure to pyrethroids, including Cypm, has been demonstrated in several environmental species [45,46], human data are very scarce. In vulnerable populations from rural areas, data indicate an increased risk of acute lower respiratory infections, like pneumonia, bronchiolitis, and acute laryngitis, in children below 5 years [47]. Better designed studies are clearly necessary to provide more robust data on potential health outcomes from exposure to pyrethroid insecticides [48].

Regarding the mechanism of action, a very old study demonstrated that Cypm (1 μM) altered the membrane lipid packing order of murine splenic lymphocytes, which may alter the stability of protein assemblies in membranes and subsequent cellular responses [49]. In line with the current knowledge and a previous study [25], the immunomodulatory effect of Cypm should be reconducted to the endocrine activity demonstrated for some pyrethroids, including Cypm. The immunosuppressive effect could be explained by the anti-androgenic activity of Cypm, as also reported by studies in the literature [50,51,52]. To evaluate the anti-androgenic profile of Cypm, we previously performed docking studies [28], which resulted in the exclusion of the competitive antagonism vs. androgen receptor (AR), and therefore an indirect anti-androgenic effect was hypothesized. In this regard, it has been demonstrated that Cypm induced the AR corepressor silencing mediator for thyroid hormone receptors and nuclear receptor corepressor recruitment, and that demonstrated that Cypm inhibited the interaction between AR and its AR transcriptional activity and to an incorrect conformation that would lead to AR degradation [53]. In line with these findings, we demonstrated that Cypm exposure was able to reduce AR expression [25]. Furthermore, Cypm was also able to reduce IL-6 release induced by LPS exposure, similarly to literature data showing that Cypm can antagonize IL-6-induced ligand-independent AR activation [54,55,56]. Therefore, the anti-androgenic activity as a Cypm mechanism of action underneath the immunosuppression was observed.

Concerning the effects of Glyp, no modulation of LPS-induced immune cell activation was observed in both models used, and if anything, the data suggest immunostimulation as evidenced by the increase in CD86 in PBMC obtained from some donors. The lack of an effect on LPS-induced proinflammatory cytokines is in agreement with data published by in human PBMC, in which Glyp (10–1000 μM) failed to modulate LPS-induced TNF-α and IL-1β production [28]. In PBMC, we previously observed a reduction of the Th1/Th2 ratio, mainly due to a decrease in Th1 cells, together with an enhancement of IL-4 and IL-17A production. Based on literature evidence that suggested Glyp being an endocrine disruptor, we also demonstrated a role of the estrogen receptor in the observed effects on Th cells [28]. Concerning the response to LPS, a potentiation rather than suppression was observed in in vivo models when mice exposed to a combination of LPS and Glyp resulted in greater cellular inflammatory effects in lungs as compared to individual exposures to LPS or Glyp [57]. The authors demonstrated that 5 days of repeated exposure to LPS + Glyp resulted in higher neutrophil counts; myeloperoxidase, TNF-α, IL-6, and creatine kinase levels; and ICAM-1 and TLR-2 expression compared to treatment with LPS or Glyp alone. After 10 days of exposure, inflammatory responses decreased; however, leukocyte infiltration persisted along with increases in IL-4 [57]. Our results do not support a modulation of LPS-induced immune activation in human cells.

## 5. Conclusions

Overall, our results contributed to the understanding of the effects by which pesticides may exert their immunotoxic effects. In addition, they may help in the future identification of specific sensitive biomarkers of pesticide-related immune alterations. Even if there is limited evidence they do, this could just reflect lack of clinical investigation. As existing epidemiological studies are inadequate to raise conclusions on immunotoxic risk associated to pesticide exposure, as several limitations apply, including poor indication on exposure levels, multiple chemical exposures, heterogeneity of the approach, and difficulty in giving a prognostic significance to the slight changes often observed, and thus further epidemiological studies are needed [7].

## Data Availability

Not applicable.
